# Viruses Hijack ERAD to Regulate Their Replication and Propagation

**DOI:** 10.3390/ijms23169398

**Published:** 2022-08-20

**Authors:** Linke Zou, Xinyan Wang, Feifan Zhao, Keke Wu, Xiaowen Li, Zhaoyao Li, Yuwan Li, Wenxian Chen, Sen Zeng, Xiaodi Liu, Mingqiu Zhao, Lin Yi, Shuangqi Fan, Jinding Chen

**Affiliations:** 1 College of Veterinary Medicine, South China Agricultural University, No. 483, Wushan Road, Tianhe District, Guangzhou 510642, China; 2 Guangdong Laboratory for Lingnan Modern Agriculture, College of Veterinary Medicine, South China Agricultural University, Guangzhou 510642, China; 3 Key Laboratory of Zoonosis Prevention and Control of Guangdong Province, Guangzhou 510642, China

**Keywords:** ERAD, viruses, viral protein, immune response, protein degradation, E3 ubiquitin ligase

## Abstract

Endoplasmic reticulum-associated degradation (ERAD) is highly conserved in yeast. Recent studies have shown that ERAD is also ubiquitous and highly conserved in eukaryotic cells, where it plays an essential role in maintaining endoplasmic reticulum (ER) homeostasis. Misfolded or unfolded proteins undergo ERAD. They are recognized in the ER, retrotranslocated into the cytoplasm, and degraded by proteasomes after polyubiquitin. This may consist of several main steps: recognition of ERAD substrates, retrotranslocation, and proteasome degradation. Replication and transmission of the virus in the host is a process of a “game” with the host. It can be assumed that the virus has evolved various mechanisms to use the host’s functions for its replication and transmission, including ERAD. However, until now, it is still unclear how the host uses ERAD to deal with virus infection and how the viruses hijack the function of ERAD to obtain a favorable niche or evade the immune clearance of the host. Recent studies have shown that viruses have also evolved mechanisms to use various processes of ERAD to promote their transmission. This review describes the occurrence of ERAD and how the viruses hijack the function of ERAD to spread by affecting the homeostasis and immune response of the host, and we will focus on the role of E3 ubiquitin ligase.

## 1. Introduction

The ER, the principal location for protein synthesis and maturation in eukaryotic cells, is rich in various molecular chaperones and enzymes that help with protein folding and modification. The host uses multiple methods to help proteins fold correctly [1]. Although all resources are devoted to protein folding, many of the nascent proteins entering the ER fail to obtain their natural conformation [2]. Eukaryotic cells regulate ER pressure mainly through the unfolded protein response (UPR) and ERAD [3,4]. UPR enhances protein folding ability by upregulating the expression of molecular chaperones and folding enzymes through a series of intracellular signal transduction responses [5], while ERAD transports the proteins that cannot be folded correctly out of the ER and further degrades them by the cytoplasmic ubiquitin-proteasome system [6,7]. Disruption of ERAD can lead to many diseases, such as Parkinson’s disease, Alzheimer’s disease, cancer, and infection, confirming its importance in correct cell functioning [8]. The genetic ablation of many ERAD components leads to the death of mouse embryos [9,10,11], which also highlights the importance of ERAD in cell and organism homeostasis. ERAD is exploited by viruses with two main features: one is that secretory proteins or membrane proteins (including many immune proteins) can be degraded by ERAD so that the virus can escape the surveillance of the immune system [12]; second, the process of ERAD transport from the ER to the cytoplasm is utilized by the virus [13], which is conducive to virus invasion. At present, many studies have revealed how viruses use ERAD. In this review, we examined the occurrence and development of ERAD and the mechanism by which different viruses use ERAD to promote its transmission.

## 2. The Occurrence and Development of ERAD

### 2.1. Substrate Recognition

**Figure 1:**
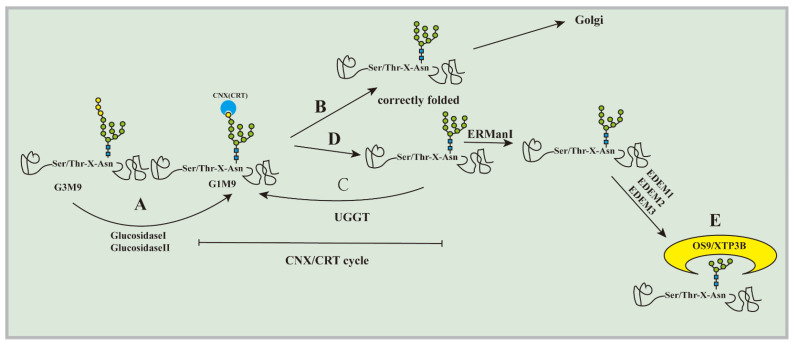
The recognition process of glycoproteins. (**A**) Glycosylation of core glycans under the action of glucosidases leads to the entry of new glycoproteins into the calnexin (CNX)/calreticulin (CRT) cycle. (**B**) The protein folded correctly and exudes to the Golgi. (**C**) Under the act of UGGT, misfolded proteins run back to interact with CNX/CRT for further folding. (**D**) With the act of ERMan\EDEM1\EDEM2\EDEM3, misfolded glycoproteins are separated from CNX/CRT to produce glycan signals for ERAD system identification. (**E**) OS-9/XTP3B recognizes misfolded glycoproteins and submits them to the next step.

Substrate recognition is the initial step of ERAD. How can the substrate of ERAD be selected from the proteins that are folded correctly or are being folded? The current understanding is still minimal. Most glycoproteins transported to the ER may cotranslate and modify a high mannose core oligosaccharide containing GlcNAc2-Man9-Glc3 (Glc: glucose, Man: mannose, GlcNAc: N-acetylglucosamine) sequence on the aspartic acid residue containing Asn-X-Ser/Thr (X represents any amino acid) at its N-terminus [14]. Glycosylation of core glycans under the action of glucosidases leads to the entry of new glycoproteins into the calnexin (CNX)/calreticulin (CRT) cycle (Figure 1A). The ‘‘CNX/CRTcycle’’ refers to the nascent N-linked glycoproteins that can bind to CNX and CRT in the ER through glucose residues. When binding to CNX and CRT, the glycoprotein starts to fold, and the folded protein with the correct structure will be released from CNX and CRT, while the protein without the correct structure will recycle and repeatedly fold until it can fold correctly [15]. For correctly folded proteins, further deglycosylation removes N-glycans to produce glycoproteins containing GlcNAc2-Man9, which prevents them from binding to CNX or CRT again, causing glycoproteins to flow out (Figure 1B). Proteins that do not have the correct conformation will be recognized by UDP-glucose: glycoprotein glucosyltransferase (UGGT). Under the action of UGGT, a glucose molecule is added to N-glycans to be glycosylated so that they can preferentially return to the CNX/CRT cycle for further folding (Figure 1C) [16,17]. Glycoproteins that cannot fold correctly must be transferred from the CNX/CRT cycle to ERAD for degradation, and mannosidases accomplish this detachment. Mannosidase removes terminal mannose residues from core polysaccharides so that they can interact with other mannose-specific lectins to combine them with ERAD [18]. Under the action of ER mannosidase I (ERManI) [19], ER degradation-enhancing α-mannosidase like protein 1 (EDEM1) [20,21], EDEM2, and EDEM3 [22,23], glycoproteins are incompatible with UGGT-mediated glycosylation, and misfolded glycoproteins are separated from CNX/CRT to produce glycan signals for ERAD system identification (Figure 1D). Soluble ER-resident proteins, OS-9 and XTP3B, play a vital role in recognizing mammalian ERAD substrates [24]. The possible mechanism for this is that OS-9 and XTP3B can recognize oligosaccharides modified by ERMan I and EDEM1-3 through the mannose-6-phosphate homologous receptor domain (MRH). The pruned oligosaccharides expose their terminal α-1 mannose and are recognized by the MRH regions of OS-9 and XTP3B. OS-9 and XTP3B bind to SEL1L through their MRH domain, and the misfolded protein is guided to the next step of reverse translocation (Figure 1E) [25,26,27]. There are many molecular chaperones involved in this process. GRP94, a metazoan-specific Hsp90 in the ER lumen, acts upstream of OS-9 to further recognize misfolded α1 subunits in a glycan-dependent manner [28].

The recognition of nonglycoproteins may be facilitated by chaperones. Folding of nonglycoproteins in the ER is assisted by the “chaperone cycle” (cycle of substrate binding and release). The ER-localized Hsp70 chaperone Bip, which is composed of the ATP-bound form and ADP-bound form, plays an important role in protein folding homeostasis [29]. Similar to Hsp70s in other compartments, Bip does so by the reversible binding and releasing of unfolded client proteins. A substrate containing a hydrophobic region exposed on the surface binds to the ATP-bound form, which is converted to the ADP-bound form by cochaperone-mediated stimulation of intrinsic ATPase activity to grab the substrate. The ADP-bound form is converted to the ATP-bound form by the action of nucleotide exchange factor (NEF) Lhs1/Grp170 to release the substrate [30]. This cycle continues until the folding is completed. Disulfide bond formation catalyzed by protein disulfide isomerase Pdi1/PDI family proteins also helps productive folding [31,32]. At present, it is known that several nonglycoproteins can be degraded through the ERAD pathway [33,34,35,36,37], and Bip plays an important role. However, how Bip plays a role in this process and how nonglycoproteins are transported to the retrotranslocation channel are still unclear.

### 2.2. Retrotranslocation and Ubiquitination

After recognition, the substrate retrotranslocates into the cytoplasm according to its specific position in the ER, which is closely combined with the ubiquitination of the substrate. In most cases, the classic E3 ligase enzyme involved in ERAD is itself a multitransmembrane protein, where the RING domain responsible for ligase activity is located in the cytoplasm, and the substrate is ubiquitinated at the same time as its reverse translocation. Subsequently, the ubiquitin substrate is extracted from the membrane in an ATP-dependent manner and released into the cytoplasm to be degraded by proteasomes. For many years, the identity of the retrotranslocation channel has been controversial. With the development of technology and the deepening of research, mainly in budding yeast but also in mammalian cells, an increasing number of components of retrotranslocation channels have been identified, and most are E3 ligase enzymes. Based on the analysis of some model substrates, the specificity of the E3 ligase enzyme complex seems to depend on the location of the misfolded lesion on the substrate relative to the ER membrane: proteins with lumen (ERAD-L substrate) or intramembrane (ERAD-M substrate) misfolded domains are targeted to HRD1 complexes; proteins with misfolded domains on the cytoplasmic side of the membrane (ERAD-C) are degraded by Doa10 complexes [38,39,40].

Based on the mutation, pull-down experiments, and interaction with the proteasome, it is speculated that the Sec61 channel, which usually allows peptides to enter the ER cavity from the cytoplasm, can work in reverse [41]. However, subsequent studies have shown that there is only a weak interaction between the ERAD substrate and Sec61, and its role as a retrotranslocation channel of ERAD has been rejected [42]. With the deepening of study, the voice of the HRD1 complex as a candidate for the retrotranslocation channel is getting louder [43], and the fact that the HRD1 complex can be used as a retrotranslocation channel of the ERAD substrate has also been proven by photocrosslinking [44]. HRD1 is a multitransmembrane protein with E3 ubiquitin ligase activity and a cytoplasmic ring finger domain that can mediate the ubiquitination of reverse transcriptional translocation substrates and act as a retrotranslocation channel. The HRD1 complex consists of the E3 ubiquitin ligase HRD1 and four other proteins (SEL1L, Der1, Usa1, and OS-9) [26,45]. SEL1L plays an important role in the HRD1 complex, and SEL1L is an important link to coordinating ERAD substrate recruitment, translocation, and ubiquitination [46]. SEL1L combines with OS-9/XTP3B [25,26], EDEM1 [47], and EDEM3 [48]. SEL1L is necessary to transfer the substrate from ER lectin to HRD1 [18,26,49]. One of the ERAD ubiquitin ligases, SEL1L, forms a nucleation complex with intact membrane ERAD components, including Derlin-1, Derlin-2, AUP1, UBXD8, VIMP, and Herp [26,27,50,51]. This, in turn, recruits the VCP/p97 complex necessary to drive substrate dislocation. The UBXD family is a diverse group of UBX (ubiquitin-regulatory X) domain-containing proteins in mammalian cells. The UBX domain enables all members of the UBXD family to bind to the multifunctional AAA-ATPase p97/VCP protein [52] via the amino terminal domain of p97 [53]. Several analyses in yeast show that to a large extent, the Hrd1-SEL1L complex itself is the retrotranslocation channel through which the substrate is brought back from the ER to the cytoplasm [54]. The self-ubiquitination of HRD1 is considered the trigger of substrate retrotranslocation, which is similar to the “door” of the substrate retrotranslocation channel [45,55,56,57,58]. Recently, the structural analysis of the HRD1 complex by cryo-EM from Saccharomyces cerevisiae showed that Hrd1 and Der1 were connected on the cytoplasmic side of the membrane through Usa1. Both Hrd1 and Der1 have a lateral door that regulates substrate entry, and these also face each other in the membrane; at the same time, Hrd1 and Der1 each have a hydrophilic groove on one side of the cytoplasm and one side of the ER, respectively. Both proteins distort the lipid membrane structure near the lateral door, making it thinner than a normal phospholipid bilayer [59].

Doa10 is located throughout the ER, including the inner and outer nuclear membranes. Localization of the Doa10 inner nuclear membrane is necessary for the degradation of its nucleoprotein substrate [60]. A recent study has shown that ubiquitin ligase Doa10 (Teb-4/MARCH6 in mammals) is a reverse transcriptase that promotes the extraction of membrane proteins. It helps to overcome the energy barrier of membrane protein extraction [61]. A recent study in yeast showed that Derlin paralog Dfm1 might also act as a core mechanism in the degradation of ERAD-M (ERAD substrates located in the membrane) [62].

In addition to the channel, the retrotranslocation of the ERAD substrate requires a force to pull them into the cytoplasm, which p97/VCP provides [63]. P97/VCP is a member of the family of ATP enzymes (AAA+ATP enzymes) related to various cellular activities, which consists of an N-terminal domain (N-domain) and two ATPase domains (D1 and D2) and contains a central pore. The ubiquitin substrate must be pulled out of the ER by the p97/VCP complex before entering the 26S proteasome for degradation [64,65]. At present, one of the generally accepted models of ERAD substrates using the p97/VCP complex is that the ubiquitin substrates are bound to the N-terminal Ufd1-Npl4 cofactor complex of p97/VCP through the ubiquitin chain and pulled into the central hole formed by the D1 domain [52], which may be exited through the sequential interaction with the monomer and the central hole of D2 [66,67,68,69]. In contrast, the C-terminal domain interacts with its adaptor proteins through the P97/VCP-binding region, P97/VCP-interacting motif, or SHP box [70]. The mechanism of this process may be that VCP provides energy for binding and hydrolysis by extracting or “shifting” the ubiquitinated substrate from the ER membrane. Ufd1, through the recognition of the K48 Ub chain, combined with the ubiquitin substrate Npl4, stabilized UFD1L [71].

### 2.3. Degradation

**Figure 2:**
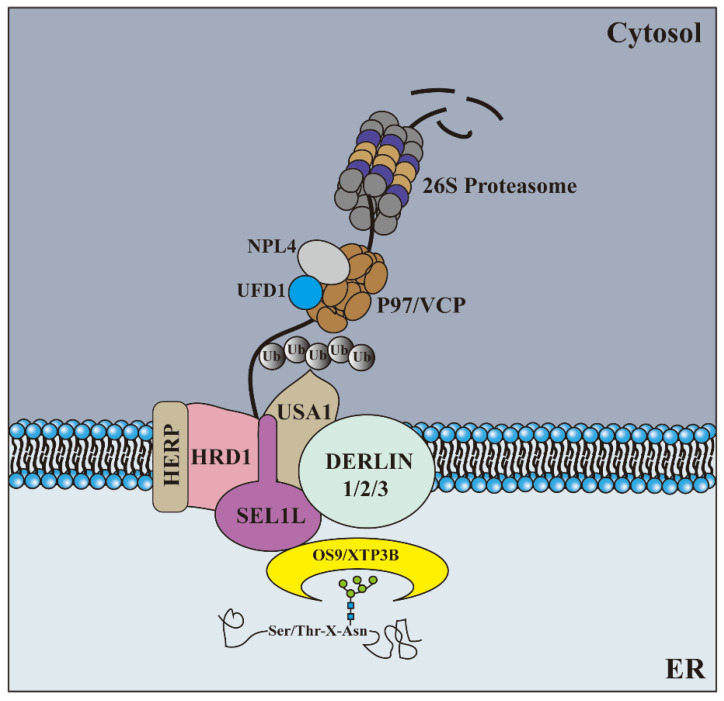
The processes of ERAD. Misfolded proteins are recognized in the ER by different quality control mechanisms, which escort terminally misfolded polypeptides to a putative channel. Cytoplasm-exposed lysine residues are ubiquitinated by ubiquitin ligases. Dislocation is completed with the help of the Cdc48p/p97 complex, and membrane-extracted substrates are conveyed to the proteasome by accessory factors.

Once misfolded proteins are identified, they must be transported to the cytoplasm where the ubiquitin-proteasome system (UPS) is located, and this process must be closely coupled with the degradation process because most of the ERAD substrates are hydrophobic and can easily accumulate in an aqueous environment. Proteins with ubiquitin- and proteasome-binding domains, such as hHR2 (Rad23 in yeast), may be used as shuttle factors to transfer the substrate from p97/VCP to the proteasome [72]. Cellular solute chaperone proteins, such as Hsc70 [73] and Bag6 [74], can interact with ERAD clients in the cytoplasm after p97/VCP-mediated extraction from the ER by exposing hydrophobic regions on these deployment clients. They may help maintain the substrate’s solubility and prevent accumulation in the aqueous cellular solute environment, but they also help the substrate guide the degradation mechanism [66]. Once transferred to the 26S proteasome, the ERAD substrate is degraded in the same manner as described by all proteins. (All the stages are shown in Figure 2, and most of the components involved in ERAD are listed in Table 1).

## 3. Viruses Hijack ERAD to Manipulate the Host Immune Response

**Figure 3:**
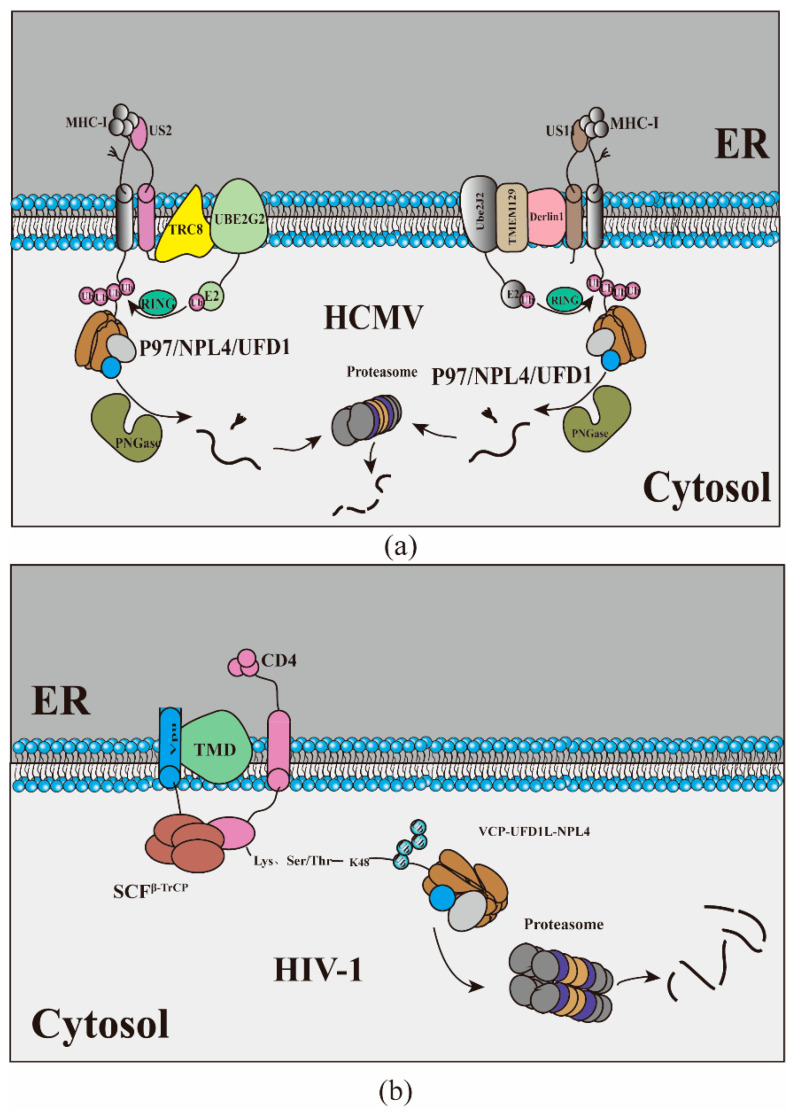
How viruses interfere with the immune system. (**a**) US11 recruits TMEM129, and TMEM129 recruits Ube2J2 for US11-induced MHC-I ubiquitin through Derlin1. US2 recruits TRC8 for US2-induced MHC-I ubiquitin. (**b**) HIV-Ⅰ targets newly synthesized CD4 receptors and rapidly degrades CD4 through ERAD.

A variety of viruses have evolved the ability to suppress the body’s immune response to promote persistent infection. Currently, the most in-depth examples of virus-induced ERAD that are involved in regulating the immune response are herpesviruses and human immunodeficiency virus (HIV). Human cytomegalovirus (HCMV) induces ERAD to accelerate the degradation of MHC-I to prevent CD8+ T lymphocytes from recognizing virus-infected cells and promoting a persistent infection. US2 and US11 are ER-resident type I membrane virus glycoproteins expressed in the early stage of HCMV infection. After cotranslation and insertion into the ER, the newly synthesized MHC-I immediately binds to US2 or US11, and US2 and US11 transport them from the ER to the cytoplasm for degradation [76]. Although both US2 and US11 can degrade MHC-I, their mechanisms do not seem to be precisely the same. US11 recruits TMEM129, and TMEM129 recruits Ube2J2 for US11-induced MHC-I ubiquitin through Derlin1; thus, driving MHC-I reverse translocation from the ER back to the cytoplasm, where MHC-I is deglycosylated by protein N-glycanase (PNGase) and then degraded by the proteasome. The E3 ubiquitin ligase TMEM129 plays a central role in US11-mediated MHC-I decomposition [77,78,79,80]. Unlike US11, which depends on Derlin-1, US2 usurps the E3 ubiquitin ligase TRC8, which plays a central role in US2-mediated MHC-I decomposition. TRC8 binds to the cytoplasmic tail of US2, resulting in rapid polyubiquitin of MHC-I, which triggers MHC-I to completely reverse into the cytoplasm [81,82]. The degradation of MHC-I was inhibited in UBE2G2-depleted cells (UBE2G2 is a kind of E2 ubiquitin binding enzyme), indicating that UBE2G2 plays a critical role in the ubiquitination of US2, possibly because of the cooperation of TRC8 and UBE2G2 [83]. Although they use different E3 ubiquitin ligases during reverse translocation, both rely on p97 to translocate MHC-I to the proteasome for degradation. In addition to MHC-I, US2 induces the downregulation of multiple immunoreceptors to modulate cellular migration and immune signaling, whereas US11-mediated degradation is restricted to MHC-I [84]. Recently, genome-wide CRISPR/Cas9 library screening found that the ubiquitin-fold modifier 1 (UFM1) pathway is also involved in the degradation of MHC-I, and interference with UFM1 specifically inhibits the reverse transport of MHC-I molecules from the ER to the cytoplasm, but the effect of UFM1 on the degradation of MHC-I molecules may be indirect [85] (For a schematic view of the mechanism, see Figure 3a). Like herpesviruses, retroviruses can also manipulate the host’s immune system through the ERAD pathway. HIV-Ⅰ Vpu targets newly synthesized CD4 receptors and rapidly degrades CD4 through a process similar to ERAD [86,87]. Magadan uses siRNA to interfere with 18 proteins related to each process of ERAD, and the results showed that the interference of VCP, UFD1L, and NPL4 had the best protection on the degradation of CD4 [71] (for a schematic view of the mechanism, see Figure 3b). Virus-infected cells are specifically eliminated through the synergistic effect of helper T cells and cytotoxic T lymphocytes. This elimination is mainly dependent on MHC-I and MHC-II presenting virus-derived peptide antigens on the surface of virus-infected cells. HCMV has evolved the mechanism to use the ERAD system to eliminate MHC-I so that the virus circumvents the monitoring of the host immune system artfully. They hijack alternative ubiquitin ligases instead of HRD1, suggesting the necessity for a specialized ubiquitin ligation system in pathogenic ERAD.

## 4. Viruses Hijack ERAD as a Transport Mechanism

**Figure 4:**
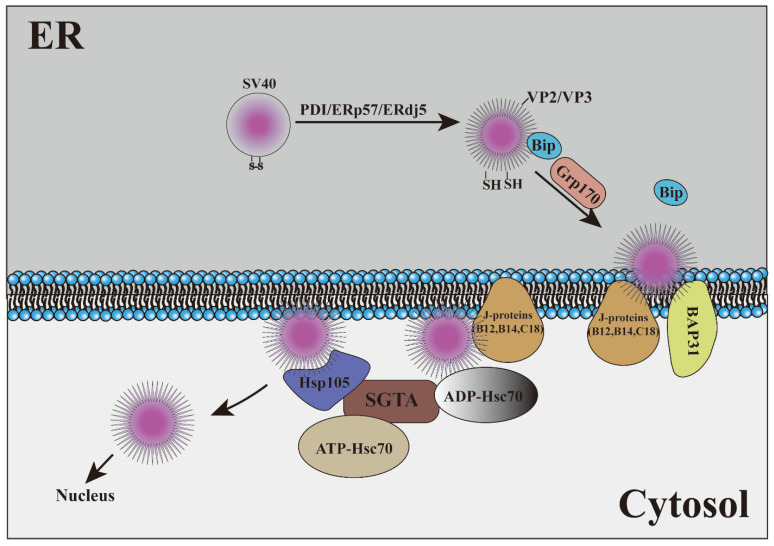
SV40 hijacks ERAD as a transport mechanism.

Studies on the use of ERAD as a transport mechanism have been reported in cholera toxins (CTx), pertussis toxins, Shiga and Shiga-like toxins (STx), heat-labile enterotoxins, Pseudomonas exotoxins (PEx), cytolethal distending toxins (CDT), and the plant toxin ricin and other toxins [88,89,90]. Some viruses have also evolved the mechanism of using ERAD to penetrate the membrane to reach the cytoplasm. Polyomaviruses (PyVs) are a kind of nonenveloped DNA tumor virus. To infect cells, PyV is transported from the cell surface to the ER, where it hijacks the elements of the ERAD mechanism to penetrate the ER and reach the cytoplasm. The virus is transported from the cytosol to the nucleus, resulting in lytic infection or cell transformation. How PyV is transported from the ER to the cytoplasm is a key issue in the process of viral infection. Many studies have shown that ERAD takes advantage of this process [91,92,93,94,95]. SV40 is the best-studied virus that utilizes ERAD as a transport mechanism. After SV40 reaches the ER, under the action of PDI, ERp57, and ERdj5, hydrophobic VP2 and VP3 proteins are exposed, and hydrophobic virus particles are produced, which are disguised as misfolded proteins [91,96,97]. To prevent the aggregation of hydrophobic virus particles, the ERAD molecular chaperone BiP was recruited by SV40 [93,98]. When the SV40-BiP complex approaches the lumen surface of the ER membrane, the virus must be released from BiP to initiate ER-membrane transport. Glucose-regulated protein, 170 kDa (Grp170), plays an important role in this process [99,100]. When hydrophobic SV40 is inserted into the ER membrane, the amino terminal region of the VP2 protein binds to the ER membrane protein BAP31 to stabilize the membrane-embedded virus and then participates in virus transport from the ER to the cytoplasm under the action of ER transmembrane J-proteins (B12, B14, and C18) [93,101,102]. Some studies have also shown that SV40 can form a specific region on the ER, which can be used as a selective membrane penetration site for viruses. The forms of VP2/VP3 exposure and membrane penetration of SV40 mainly exist in these specific regions, and SV40 mutants that cannot be transferred across the ER membrane to the cytoplasm cannot trigger the formation of lesions [92,103]. The transfer of SV40 from the ER to the cytoplasm also requires a “force”. In general, the driving force of the ERAD process is usually provided by p97, but studies have shown that this “force” may be provided by Hsc70-Hsp105-SGTA rather than p97 in SV40 [92]. Under the action of membrane-bound J proteins, Hsc70 was transformed into high-affinity ADP-Hsc70. This enables Hsc70 to first bind to membrane-embedded SV40, and Hsp105 then induces the nucleotide exchange of Hsc70 to produce ATP-Hsc70 that releases viral particles. Once the virus is detached from Hsc70 and Hsp105, it can capture SV40 and release it from Hsp105, with Hsc70 recombining the virus. The iterative cycles of Hsc70–Hsp105 binding and releasing SV40 are considered to provide the main driving energy for extracting viral particles from the ER membrane [104,105,106,107]. In short, the unenveloped virus SV40 transports the virus from the ER to the cytoplasm through the host ERAD pathway and inserts the virion into the ER by changing the structure of the virion (Figure 4). The formation of a unique membrane penetration site in the local cell membrane so that the virus can successfully enter the cytoplasm is a new discovery of the virus in the process of using ERAD.

## 5. Viruses Hijack ERAD to Regulate Viral Protein Expression

ERAD plays an essential role in the regulation of viral protein expression. JEV and DENV degrade NS4B protein in convoluted membranes (CM) through the Derlin2-mediated ERAD pathway to avoid the excessive accumulation of nonstructural proteins from damaging virus replication. In addition, the researchers also found that JEV and DENV NS4B proteins can interact with p97/VCP. The inhibition of VCP activity by VCP-specific chemical inhibitors not only inhibited the degradation of nonstructural proteins but also inhibited the production of infectious JEV and DENV virus particles [108]. A recent study showed that Bardoxolonemethyl (CDDO-me), an inhibitor of protein translocation mediated by HRD1, can bind to grp94, a vital component of the HRD1 pathway, thus inhibiting the replication of DENV and ZIKV. The knockdown of grp94 can also significantly inhibit the replication of DENV and the synthesis of viral membrane proteins [109]. Ruan found that CP26, another small molecular inhibitor that targets the HRD1 complex and inhibits the process of protein dislocation from the ER cavity to the cytoplasm, can also inhibit DENV and ZIKV infection in cells [110]. HCV, a member of Flaviviridae, can also activate ERAD after.

Infection studies have shown that HCV infection induces the expression of EDEM1, EDEM2, and EDEM3. When IRE1 was knocked out, the increase in XBP1 splicing and EDEM induced by HCV was reversed, indicating that HCV-induced ERAD is caused by IRE1. Subsequent studies showed that EDEMs could interact with HCV E1 and E2 proteins, which significantly increased the ubiquitination of HCV glycoproteins and significantly improved the stability of HCV E2 proteins after EDEM1 knockout, and EDEM knockout can significantly promote the production of HCV infectious particles but does not affect the replication of the HCV genome [48], which may play a role by controlling the posttranslational modification of HCV glycoproteins. Similar to HCV, the HBV envelope protein can also be degraded through ERAD. HBV infection upregulates the level of EDEM, especially EDEM1, which is the result of viral protein expression, independent of viral replication and nucleocapsid protein expression, and EDEM1 can interact with the HBV envelope protein. To study whether EDEM1 can affect the stability of envelope proteins, wild-type S, M, and L envelope proteins were cotransfected with the EDEM1 plasmid or siRNA in 293T cells. The results showed that the overexpression of EDEM1 promoted the degradation of the HBV envelope protein, and the interference of EDEM1 promoted the stability of the viral envelope protein, thus controlling the number of natural peptides available for SVPs assembly and virion envelope [111]. Wang found that the level of SEL1L in the liver of HBV carriers was significantly higher than that within immune tolerance. Overexpression of SEL1L in cells leads to a significant decrease in the levels of HBV, RNA, DNA, core, and envelope proteins, and the silencing of SEL1L leads to the opposite result. However, the decrease in RNA mediated by SEL1L is not due to transcriptional inhibition but may be related to a posttranscriptional mechanism. Treatment with inhibitors of ERAD significantly increased the level of intracellular S protein but did not increase the level of envelope protein, indicating that the decrease in the core protein caused by SEL1L overexpression was not through the ERAD pathway [112].

UL148 is a viral ER (ER)-resident glycoprotein that contributes to the cellular tropism of human cytomegalovirus (HCMV). The effect of UL148 on tropism is related to its potential to promote the expression of glycoprotein O (gO). To study whether UL148 regulates gO abundance by affecting ERAD, small interfering RNA (siRNA) silencing was performed on SEL1L or its partner HRD1 in the case of an infection. The knockout of these ERAD factors will significantly increase the level of gO but will not increase the level of glycoproteins from other viruses, and the effect is amplified in the presence of UL148. Further studies have shown that UL148 can interact with the ERAD adapter SEL1L in the infected state. In addition, the pharmacological inhibition of ERAD showed similar results. The silencing of SEL1L during infection also stabilized the interaction between gO and ER lectin OS-9, which also indicated that gO was the substrate of ERAD [113]. The posttranslational instability of viral glycoproteins provides a basis for the mechanism of viral regulation of tropism and transmission.

Env is a necessary protein for HIV-Ⅰ to enter cells, and HIV-Ⅰ Vpr stabilizes HIV-Ⅰ Env glycoprotein by preventing the degradation of Env through the lysosomal pathway [114]. The mechanism may be that the Vpr protein enhances the redox state in the ER and promotes the folding of Env [115]. The replication of HIV-Ⅰ was severely inhibited in the human CD4+ T-cell line CEM. NKR (NKR). Zhou suggested that this phenomenon may be because mitochondrial translocator protein (TSPO) can induce the rapid degradation of the HIV-Ⅰ envelope (Env) through the ERAD pathway in NKR cells [116]. ERManI inhibited the expression of HIV-Ⅰ Env in a dose-dependent manner. The knockout of ERManI interferes with TSPO-mediated Env degradation. Further studies show that ERManI can interact with Env, and its catalytic domain and enzyme activity play a key role in the degradation of Env [117].

When the Flaviviridae virus replicates, it first synthesizes polypeptide-containing structural proteins and nonstructural proteins and then cleaves these into single structural proteins and nonstructural proteins under the action of an enzyme. The infected cells produce the same number of structural and nonstructural proteins, but the virions are mainly composed of structural proteins, and the excessive accumulation of nonstructural proteins will damage the replication of the virus genome. Viral structural proteins and nonstructural proteins need to be in a state of balance, and the homeostasis of this viral protein level is considered necessary for the stable transmission of the virus [118]. Most viruses can control the amount of each viral protein at the transcriptional level; however, viruses of the Flaviviridae and Picornaviridae families do not have such properties. Recent studies have proposed a model suggesting that the cellular ERAD pathway plays a central role in maintaining viral protein homeostasis. That is, Flaviviridae may have evolved to usurp the ERAD system to discard extra NS proteins in virus-infected cells.

## 6. Viruses Utilize EDEMosomes as an Enclosed Safe Scaffold for Their Replication

Under normal growth conditions, the activity of the ERAD mechanism must be kept at a low level to avoid the premature interruption of folding procedures and to obtain natural structures rather than degrade immature peptides [119]. The ERAD process may be regulated or tuned through the disposal of its own regulating factors through proteasomes or autophagosomes/vesicular trafficking to lysosomes [120]. It relies on the selective sorting of EDEM1 and some other short-lived ER chaperones that are carried by 200–800 nm vesicles called EDEMosomes. Some evidence shows that there is LC3/Atg8 at the EDEMosome limiting membrane [121]. LC3 plays a crucial regulatory role in autophagy; when autophagy occurs, LC3-I is converted into LC3-II and located in autophagosomes. Interestingly, unlike autophagosomes, LC3-positive EDEMosomes do not contain LC3-II; rather, LC3-I is noncovalently associated with its limiting membrane.

Some positive-strand RNA viruses can hijack ERAD as well. Coronavirus induces the formation of double-membrane vesicles (DMVs), CMs, and open double-membrane spherules (DMSs) in the process of virus replication, providing sites for virus replication, transcription, and translation and avoiding recognition by the host immune system [122,123]. EDEMosomes provide an appropriate enclosed structure. Recent studies have found that EDEM1, OS-9, and SEL1L colocalize with mouse hepatitis virus (MHV) double-stranded RNA and viral protein nsp2/3 (nonstructural proteins 2 and 3), which are part of replication and transcription complexes [121]. The siRNA-mediated knockdown of SEL1L receptor cells can reduce the acceleration of MHV transmission [124]. This suggests that MHV hijacks ERAD to regulate vesicle replication. Subsequently, it was reported that equine arteritis virus (EAV) hijacked ERAD regulatory vesicles like MHV, indicating that this mechanism may be conservative in different viral strains of Nidovirales [125]. Cells infected with SARS-CoV also showed a significant accumulation of EDEM1 [121]. Electron tomography showed that NSP-3 and NSP-4 were coexpressed in SARS-CoV to induce DMV vesicles. In addition, many DMVs and CMs were found near the nuclei of Huh7 cells infected by MERS-CoV [124,125]. The consequence of coronavirus infection in the ERAD pathway induces the formation of an encapsulated bilayer membrane covered by LC3-I; although autophagy is not necessary for MHV infection, the loss of LC3 will prevent the formation of DMV. Some studies have shown that the deletion of the autophagy key genes Atg5 and Atg7 will not effectively respond to virus replication; even in the case of blocking autophagy, MHV can maintain its transmission ability [125]. However, the depletion of LC3-I leads to a significant decrease in viral load. The same is true in JEV. The inhibition of JEV replication has been observed by inhibiting the expression of SEL1L and EDEM1. In fact, JEV cannot restore its proliferation without releasing EDEMosomes [126]. However, it is still unclear how DMVs are shaped from single-membrane EDEMosome vesicles.

## 7. Conclusions

As a molecular mechanism for the degradation of misfolded proteins, ERAD is highly conserved in eukaryotes and controls the disposal of misfolded or misassembled proteins. Deregulation of this process can lead to pathogenic conditions. Because ERAD can be used as a method for protein degradation, during the game between the virus and the host, the virus has evolved a mechanism to degrade MHC-I or virus proteins through ERAD; it helps the virus escape immune surveillance to achieve sustained infection or balance the number of viral proteins to promote viral replication. As this degradative pathway contains a portal for a protein to reach the cytosol from the ER, it can be co-opted by viruses to gain access to the host’s cytosol during infection. Our review analyzed how well-characterized viruses hijack elements of the ERAD pathway to accomplish this feat (see Table 2) and focused on the role of E3 ubiquitin ligase. As a vital site for protein biosynthesis, the ER plays a basic role in maintaining cellular integrity, but ironically, the virus can target the organelle to provide favorable conditions for virus replication.

Several questions remain to be answered. First, although there is evidence of a crucial role for ERAD within virus development, the precise mechanism and identification of ERAD substrates in this process are still limited. Second, ERAD is an important measure to maintain the homeostasis of ER, but current studies have shown that the ERAD process can be used by viruses to promote viral replication, and the mechanism of how viruses manipulate ERAD is still limited. Third, can the ERAD pathway be modulated pharmacologically to prevent viral infection? The role of ubiquitin in virus infection is an intriguing new topic with great potential to provide new insights into the host machinery involved in regulating virus infection. We presented examples of viruses belonging to a broad number of virus genera that interact with the E3 ubiquitin ligase to either regulate its own replication cycle or evade host defenses. The discovery of more E3 ubiquitin ligases involved in ERAD will deepen our understanding of the ERAD process to screen out suitable antiviral drugs. As an aggregate, this work shows that viruses can manipulate the ERAD machinery to suppress host defenses, modulate virus replication, and regulate viral protein turnover.

## Figures and Tables

**Table 1:** List of components involved in ERAD.

Component (Yeast)	Component (Mammals)	Function	References
Kar2	Bip	Substrate recognition and recruitment	[75]
Cne1	Calnexin (CNX)	Lectin chaperone	[14,15]
	Calreticulin (CRT)		
-	UGGT1	Glycoprotein glucosyltransferase	[17]
	UGGT2		
Mns1	Man1B1 (ERMan Ⅰ)	N-glycan trimming from M9	[19]
Htm1	EDEM1	N-glycan trimming fromM8 to M7	[20,21]
	EDEM2	N-glycan trimming fromM9 to M8	[23]
	EDEM3	N-glycan trimming fromM8 to M7	[22,23]
Yos9	OS-9	Recognize a terminal α1,6-linked mannosyl residue	[24]
	XTP3		
Hrd1	HRD1	Retrotranslocation channel	[44,59]
	gp78		
Hrd3	SEL1L	Substrate recognition and recruitment	[46]
Der1	Derlin1	Retrotranslocation channel	[59]
	Derlin2		
	Derlin3		
Doa10	Teb-4/MARCH6	Retrotranslocation channel	[60]
Cdc48	p97/VCP	Substrates dislocation	[63]
Ufd1	UFD1	Cofactor of p97	[52]
Npl4	NPL4	Cofactor of p97	[52]

**Table 2:** Mechanism by which viruses hijack ERAD.

Virus	ERAD Component	Mechanism	References
HCMV	TMEM129, Derlin1, Ube2j2	US11 recruits TMEM129, and TMEM129 recruit Ube2J2 driving MHC-I to cytoplasm, then deglycosylated by PNGase	[77,78,79,80]
	TRC8, Ube2g2	TRC8 bind to US2, resulting in polyubiquitin of MHC-I,	[83]
HIV	VCP, UFD1 L, NPL4	Vpu targets CD4 receptors and rapidly degrades CD4	[86,87]
SV40	PDI, ERp57, ERdj5	SV40 VP2 binds to BAP31 to stabilize the membrane-embedded virus and then SV40 is transport to the cytoplasm under the action of ER transmembrane J-proteins	[98,101,102]
DENV	Derlin2, grp94, VCP	avoid excessive accumulation of nonstructural protein	[108,109,110]
ZIKV	HRD1	avoid excessive accumulation of nonstructural protein	[109,110]
JEV	VCP	avoid excessive accumulation of nonstructural protein	[108]
HCV	EDEM1, EDEM2, EDEM3	IRE1 induces ERAD to degrade nonstructural protein	[48]
HBV	EDEM1, SEL1L	degrade nonstructural protein	[111,112]
MHV	EDEMosome	nsp2 and nsp3 make RTC near to ER and induces EDEMosome, DMV, CM and DMS	[121,124]
EAV	EDEMosome	utilize EDEMosome as replication sites	[125]
SARS-CoV	EDEMosome	nsp3/4 induces DMV construct	[124,125]

## Data Availability

Not applicable.
